# Analgesic strategies aimed at stimulating the endogenous production of allopregnanolone

**DOI:** 10.3389/fncel.2014.00174

**Published:** 2014-06-17

**Authors:** Pierrick Poisbeau, Anne Florence Keller, Maya Aouad, Nisrine Kamoun, Ghislaine Groyer, Michael Schumacher

**Affiliations:** ^1^Molecular Determinants of Pain, Institute for Cellular and Integrative Neurosciences (INCI), UPR Centre National de la Recherche Scientifique (CNRS) 3212 and University of StrasbourgStrasbourg, France; ^2^Rhenovia PharmaMulhouse, France; ^3^UMR 788 Neuroprotection and Neuroregeneration: Neuroactive Small Molecules, Institut National de la Santé et de la Recherche Médicale (INSERM) and University Paris-SudKremlin-Bicêtre, France

**Keywords:** allodynia, hyperalgesia, nociception, pain, neurosteroids, etifoxine

## Abstract

A growing number of studies indicate that 3-alpha reduced neurosteroids are remarkable analgesics in various pain states. This is the case for allopregnanolone (AP), one of the most potent endogenous positive allosteric modulators of GABA_A_ receptor function. From the pioneering work of Hans Selye, who described the sedative properties of steroids, synthetic compounds resembling the progesterone metabolite AP have been developed. If some of them have been used as anesthetics, it seems difficult to propose them as a therapeutic option for pain since they display several adverse side effects such as sedation, amnesia and functional tolerance. An alternative strategy, chosen by few laboratories around the world, is aimed at stimulating the local production of 3-alpha reduced neurosteroids in order to limit these well-known side effects. This pharmacological approach has the advantage of targeting specific structures, fully equipped with the necessary biosynthetic enzymatic machinery, where neurosteroids already act as endogenous pain modulators. The various pharmacological trials which attempted to treat pain symptoms by stimulating the production of 3-alpha reduced neurosteroids are reviewed here, as well as novel neurotransmitter systems possibly regulating their endogenous production.

## Introduction

In 1941, Selye reported that intraperitoneal injections of high doses of progesterone produce anesthesia in the rat (Selye, 1941). Several years later, this led to the development of steroidal anesthetics (Child et al., 1971). Of particular interest are the 3α-reduced steroid compounds, such as alphaxalone, which display potent anesthetic properties. Indeed, they were found to selectively act as positive allosteric modulators of the inhibitory functions of GABA_A_ receptors (GABA_A_Rs), expressed either at extrasynaptic (Harrison and Simmonds, 1984) or synaptic sites (Poisbeau et al., 1997; Cooper et al., 1999). After two decades of research, two steroid binding sites on GABA_A_Rs have been identified (Hosie et al., 2006). Modulation of GABA_A_R function is observed after binding of a 3α-reduced steroid in a cavity formed by the α-subunit transmembrane domains. A direct activation of the receptor-channel is also observed at a higher concentration if the binding is effective at interfacial residues between α and β subunits. Interestingly, recent experiments strongly suggest that these binding sites are reached by steroids via lateral mobility in the cell membrane, and that they only affect GABA_A_R function when accessing the intracellular part of the channel (Akk et al., 2005). This observation may help understanding the possible occlusion of steroid action when the receptor is submitted to intracellular phosphorylation by protein kinase C (Harney et al., 2003; Vergnano et al., 2007). Many other receptor-channels were found to be modulated by steroids (Schlichter et al., 2006) but, in most cases, this physiological action was only observed at micromolar concentrations. Without fully excluding elevated levels of steroids within specific neuronal microdomains, such concentrations are unlikely to occur in the central nervous system (CNS; Schumacher et al., 2003).

**Figure 1:**
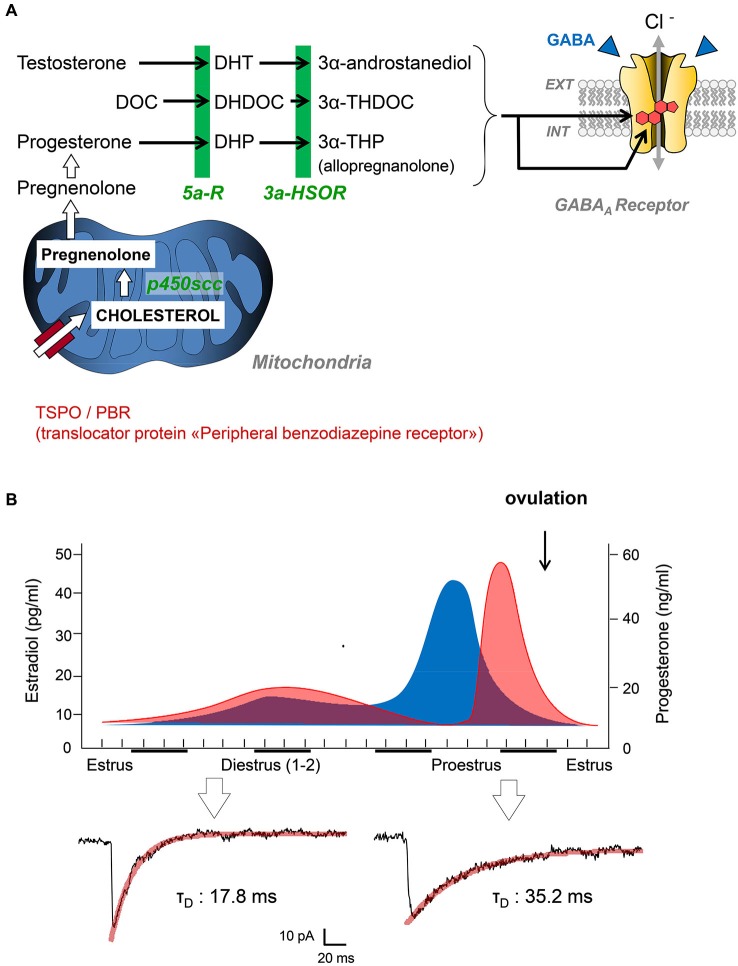
**(A)** Simplified diagram summarizing the key steps of neurosteroidogenesis from cholesterol precursors to 3α-reduced neurosteroids (3α-androstanediol, 3α-THDOC, 3α-THP). These end-chain metabolites are potent positive allosteric modulators of GABA_A_Rs after binding to specific binding sites. Abbreviations: DOC, deoxycorticosterone; DHDOC, 5α-dihydrodeoxycorticosterone; THDOC, 3α,5α-tetrahydrodeoxycorticosterone; DHP, 5alpha-dihydroprogesterone; THP, 3α,5α-tetrahydroprogesterone (= AP); 5α-R, 5α-reductase; 5α-HSOR, 3α-hydroxysteroid oxydoreductase; P450scc, P450 side-chain cleavage enzyme. **(B)** Top graphs summarize the global changes in blood estradiol (blue) and progesterone (red) during different phases of the reproductive cycle in female rats. Patch clamp traces below are representative of inhibitory postsynaptic currents mediated by GABA_A_Rs in the spinal cord of female Sprague-Dawley rats during estrus and proestrus (i.e., when progesterone levels are low and high, respectively). Adapted from Keller (2002).

It is interesting to note at this stage that neuroactive steroids can be synthesized at the periphery (i.e., by gonads and adrenal glands), but also by neural cells in the nervous system. This discovery led Baulieu and collaborators to propose the term “neurosteroids” for neuroactive steroids produced by neural cells independently of the endocrine steroidogenic glands (Baulieu and Robel, 1990). In the brain, similarly to any steroid-synthesizing tissues, a mitochondrial protein complex called TSPO is necessary to initiate the synthesis of neuroactive steroids (Rupprecht et al., 2010). TSPO facilitates the translocation of cholesterol from the outer to the inner mitochondrial membrane, where the P450 side-chain cleavage enzyme is located. The intramitochondrial transport of cholesterol is considered as a rate-limiting step for neurosteroidogenesis. Of course, this complex metabolic step can be bypassed if peripheral steroid hormones access the brain, as they easily cross the blood-brain barrier. In this case, circulating steroid hormones (i.e., progesterone, deoxycorticosterone, testosterone) may serve as precursors for neurosteroidogenic enzymes synthesizing GABA_A_R active steroids (Figure 1A).

The purpose of this topical review is to illustrate the current analgesic strategies aimed at stimulating the production of endogenous neurosteroid analgesics in animal models. Two major aspects will be covered: (i) the recent attempts to use TSPO agonists for the production of steroid analgesics; and (ii) the identification of endogenous signaling pathways which may regulate neurosteroidogenesis and could be targeted in the near future for therapeutic purposes.

## What are the endogenous steroid analgesics? Lessons from exogenously-administered steroids and current hypothesis for sex-specific pain issues

Since the early reports of Selye, several groups have characterized the analgesic efficacy of steroid hormones, when administered systemically, into cavities of the CNS (intracerebroventricular, i.c.v. or intrathecally) or directly into rodent brain structures. These observations also raised critical issues linked to the well-known sex-specific differences in pain responses (Greenspan et al., 2007). From many experiments, it is clear that the administration of testosterone or progesterone to rodents induces antinociception and analgesia (Kavaliers and Wiebe, 1987; Frye and Duncan, 1994; Pednekar and Mulgaonker, 1995). Conversely, low levels of theses hormones, after gonadectomy for example, are associated with low nociceptive thresholds and pain hypersensitivity. Experimental evidences have also shown that the effects of testosterone and progesterone are mediated by their neuroactive metabolites, since analgesia is never observed in mice deficient for 5α-reductase (Frye et al., 2002, 2004). We recently contributed to this field by showing that the analgesic action of AP (3α-hydroxy-5α-pregnan-20-one or 3α,5α-tetrahydroprogesterone) is mediated by a direct allosteric positive modulation of GABA_A_Rs in the spinal cord of rats displaying mechanical or thermal pain symptoms (Charlet et al., 2008). Coming back to the endocrine control of nociception by steroid hormones, it is interesting to note that the duration of miniature GABA_A_R-mediated synaptic currents in the spinal cord is submitted to large variations during the female reproductive cycle (Figure 1B). We found these currents to be significantly longer in duration while recording from layer II spinal neurons of adult females during proestrus (i.e., during the progesterone surge) compared to those recorded in late estrus when circulating progesterone is low (mean decay time constant in proestrus: 31.9 ± 3.3 ms, *n* = 10 vs. estrus: 16.8 ± 2.9, *n* = 14; two-tailed unpaired Student’s *t*-test, *p* < 0.001). Interestingly, glycinergic synapses during proestrus are also converted into mixed glycine/GABAergic synapses. Together, this led to an increased inhibitory control of layer II neurons processing pain informations and in a reduction in the intensity of pain symptoms, as previously demonstrated (Keller et al., 2001, 2004; Inquimbert et al., 2008). Since layer II neurons are fully equipped to synthesize AP from progesterone, it is likely that progesterone reaching the spinal cord is converted to AP, which potentiates the affinity of GABA_A_Rs for GABA at individual synapses. This is fully in agreement with previous studies indicating that female pain thresholds are controlled by the levels of circulating gonadal steroids and, among them, progesterone (Greenspan et al., 2007).

Based on these observations, several groups around the world, including ours, have demonstrated the efficacy of administering AP for alleviating pain symptoms (Kavaliers and Wiebe, 1987; Frye and Duncan, 1994; Charlet et al., 2008; Meyer et al., 2008, 2011). This strategy, although very efficient in the short-term, has major disadvantages since a systemic/oral administration of AP-like steroids may generates negative side effects such as sedation, fatigue, nausea and functional tolerance. If the treatment is stopped or not properly controlled, it may also give rise to severe withdrawal symptoms (Smith, 2001, 2002; Gulinello and Smith, 2003). It remains that AP has an interesting therapeutic potential as pain killer in pathological pain states. Interestingly, endogenous concentrations of AP are affected in various neuropathologies including peripheral neuropathies (Melcangi et al., 2011). For example, AP concentrations were found particularly low in the distal portion of an injured sciatic nerve and correlated with low expression levels of 5α-reductase (Roglio et al., 2008). In apparent contradiction, 5α-reductase activity is high in the spinal cord of rats exhibiting inflammatory pain symptoms (Poisbeau et al., 2005). Since pain responses are exacerbated after inhibition of 5α-reductase activity, this demonstrates that the synthesis of AP-like steroids may limit pain symptoms. More recently, 3α-HSOR expression and activity were also found to be elevated in the spinal cord dorsal horns of neuropathic rats (Meyer et al., 2008).

Taken together, these results show that AP-like compounds, exogenously administered, are particularly efficient for limiting pain symptoms. Several reports also indicate that they may exert a general neuroprotective action. In peripheral nerves, in the spinal cord and in various supraspinal structures, there is a significant production of these putative pain killers (Caruso et al., 2013). A straightforward strategy would be to stimulate this widespread endogenous analgesic system. This has, at least, one major advantage: to limit the possible unwanted side effects seen when AP is given *per os* or via the general circulation.

## Stimulating the synthesis of allopregnanolone for producing analgesia

The mitochondrial TSPO complex is the main molecular target exploited so far to efficiently stimulate neurosteroidogenesis (Rupprecht et al., 2009). Many TSPO ligands have been developed and tested successfully as neurotherapeutics in experimental models of painful diabetic and chemotherapy-induced neuropathy (Bordet et al., 2008; Aouad et al., 2009), mononeuropathy after chronic nerve constriction (Aouad et al., 2014a) and monoarthritis induced by persistent knee inflammation (Aouad et al., 2014b).

When we first attempted to stimulate the production of 3α-reduced neurosteroids in the spinal cord, we incubated the slices in the presence of the classical benzodiazepine diazepam and of flumazenil, a silent antagonist of the benzodiazepine site on GABA_A_Rs (Keller et al., 2004). This favored the binding of diazepam on the mitochondrial TSPO and promoted the local synthesis of AP-like neurosteroids. Indeed, GABA_A_R-mediated synaptic currents were longer in duration, suggesting an increased affinity of the receptor-channel for GABA. We further confirmed 3α-reduced neurosteroids to be responsible for this increased spinal inhibition by blocking this effect with the TSPO inhibitor PK11195 or the 3α-reductase inhibitor finasteride (Keller et al., 2004). Apart from characterizing the key role of neurosteroids in the developmental maturation of inhibitory spinal synapses, the increased spinal inhibition was found to be responsible for the limitation of thermal hyperalgesia in a rodent model of inflammatory pain (Poisbeau et al., 2005). Beside GABA_A_Rs, note here that 3α-reduced neurosteroids may also inhibit T-type calcium channels to produce analgesia as previously published (Pathirathna et al., 2005). As mentioned earlier, it is difficult to use exogenous administration of AP-like steroids or benzodiazepines *in vivo* (see however Brinton, 2013). We thus tried to use a TSPO ligand to achieve similar goals. We choose etifoxine (EFX) because its properties had been already characterized on primary culture of hypothalamic neurons and on freshly-dissociated neonatal spinal neurons (Schlichter et al., 2000).

EFX (2-ethylamino-6-chloro-4-méthyl-4-phényl-4H-3,1-benzoxazine chlorhydrate) is prescribed in several countries as a non-benzodiazepine anxiolytic (Servant et al., 1998; Nguyen et al., 2006). At an efficient anxiolytic dose in human, it can be used safely (e.g., no functional tolerance and no physical dependence after treatment cessation) and displays limited adverse side effects on cognitive functions and vigilance (Micallef et al., 2001). EFX preferentially binds and modulates GABA_A_Rs containing β2/3 subunits, at a site close to the chloride channel and distinct from that of benzodiazepines (Verleye et al., 1999, 2002; Hamon et al., 2003). Beside this direct effect on GABA_A_Rs, EFX also binds to TSPO with an apparent affinity of about 20 μM (Verleye et al., 2005). If rats are sacrificed 30 min after a single injection of EFX of the reference anxiolytic dose of 50 mg/kg i.p., the plasmatic and brain concentrations of AP are increased by 2–4 times (Verleye et al., 2005). An increase in AP was also seen in EFX-treated male rats in the absence of gonads and adrenal glands. Because AP is a potent allosteric positive modulator of GABA_A_Rs, this molecular mechanisms is referred to as “indirect” on GABA_A_R function. While studying the functional consequence of EFX on hypothalamic primary cultures, we found that it strongly affected the tonic inhibition mediated by extrasynaptic receptors (Schlichter et al., 2000). More recently, we also found that the indirect neurosteroid-mediated effect of EFX prolonged GABA_A_R-mediated synaptic currents in layer II neurons of the spinal cord (Aouad et al., 2014b).

**Figure 2:**
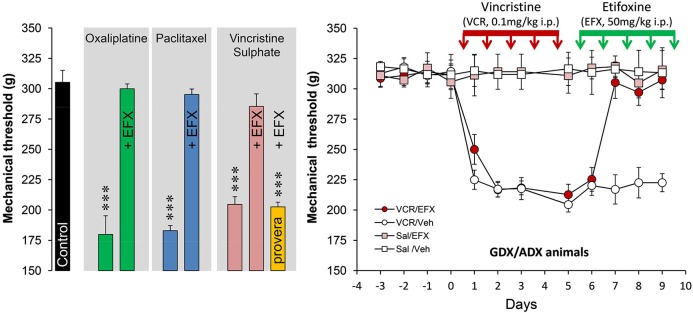
**Histogram on the left illustrates the mechanical nociceptive threshold measured with a calibrated forceps in control (black bar, treated with the vehicles) and after treatment with oxaliplatin (green bars; 2 mg/kg, injection i.p. twice-weekly for four-and-a-half consecutive weeks), paclitaxel (blue bars; 2 mg/kg i.p. on four alternate days: day 1,3,5,7), vincristine sulphate (pink bars; 0.1 mg/kg, five daily injections i.p.)**. Note that all rats exhibited low mechanical thresholds after chemotherapy (Tukey, *p* < 0.001; *n* = 6–8 rats per groups) but normal thresholds were restored after five daily injections with etifoxine (EFX, 50 mg/kg i.p.). EFX analgesia was abolished in animals pre-treated for 1 week before VCR with depo-provera (orange bar; inhibitor of 3α-HSOR, five daily injections of 5 mg/kg s.c.). Graph on the right illustrates the time course of mechanical threshold in gonadectomized/adrenalectomized (GDX/ADX) rats, submitted to VCR chemotherapy (or saline) and treated for their mechanical allodynia with EFX (or vehicle). Note that EFX analgesia persisted even in the absence of peripheral source of steroids. Abbreviations: EFX: etifoxine, Sal: saline, VCR: vincristine, Veh: vehicle. Adapted from Aouad et al. (2010).

Spinal inhibition, when reduced, gives rise to pathological pain symptoms and it is then crucial to maintain or to increase inhibitory controls to limit pain states. Because EFX may theoretically increase inhibitory controls mediated by GABA_A_Rs, directly or indirectly, we choose to administer EFX (50 mg/kg, five daily injections i.p.) to animals exhibiting generalized neuropathic pain symptoms after chemotherapy with vincristine (VCR) sulphate, oxaliplatin or paclitaxel (Figure 2). The very low values for mechanical thresholds after chemotherapy were consistent with the presence of mechanical allodynia in all rat groups and models. Normal mechanical thresholds were, however, restored after EFX treatment, except if rats were pre-treated with an inhibitor of 3α-HSOR (depo-provera = medroxyprogesterone acetate) as shown in Figure 2 (orange bar). Interestingly, EFX analgesia persisted even if the gonads and adrenals were removed (Figure 2, right graph). This strongly suggests that EFX analgesia is almost exclusively carried by the production of AP-like neurosteroids.

## TSPO expression, possible cell cooperation and signaling mechanisms

The above results, combined with recently published data (Aouad et al., 2010, 2014a,b), raise several critical questions regarding the TSPO-mediated mode of action of EFX. First, EFX clearly displays anti-allodynic/hyperalgesic properties but does not modify basal nociceptive thresholds of symptom-free animals (or body parts; e.g., sham-operated paw or contralateral territories to the lesion site). The second important observation is related to the efficacy of the effect, which not only fully alleviates pain symptoms, but also prevents their re-appearance after cessation of the treatment. In the model of rat sciatic nerve constriction, we failed to observe any pain symptoms for about 90 days after the treatment (Aouad et al., 2014a). As highlighted in a Pain editorial, there is thus an urgent need for a clinical trial testing the translational interest of this molecule in human pain pathologies (Zeilhofer, 2009). In addition, the molecular and cellular mechanisms recruited by EFX need to be identified and will require complementary experiments. Beside the sensori-discriminative component of pain responses, the real action of EFX on the affectivo-emotional component need, for example, to be clarified. This will require studying the supraspinal action of EFX in brain structures processing nociceptive informations and setting the emotional pain responses.

Of particular interest are the neuroregenerative and neuroprotective effects of the molecule described in a model of sciatic nerve lesion (Girard et al., 2008) or of diabetic neuropathy (Giatti et al., 2009). After cryolesion of the rat sciatic nerve, EFX therapy not only accelerated but also improved the quality of axonal regeneration and functional recovery (Girard et al., 2008). This was an important observation, as poor regeneration of axons may result in chronic neuropathic pain. Once again, little is known regarding the molecular mechanisms recruited to protect and promote peripheral nerve recovery, and there is so far little information concerning the cellular targets of EFX. Within the peripheral nervous system, TSPO is upregulated in macrophages, Schwann cells and dorsal root ganglia (DRG) sensory neurons in response to injury and disease (Karchewski et al., 2004; Rupprecht et al., 2010). A synergistic cooperation between these different cell types could be of utmost importance for neuroregenerative processes as well as pain-related neuropathologies.

Indeed, TSPO protein levels are upregulated in the ipsilateral spinal cord in rats displaying inflammatory pain symptoms after knee injection of complete Freund’s adjuvant (Hernstadt et al., 2009). A recent study also reports an increase in the number of TSPO binding sites (e.g., using [^3^H] PK11195, a well known TSPO ligand) in the spinal cord of rats, exhibiting neuropathic and osteoarthritic pain symptoms (Miller et al., 2013). According to this study, the increase in [^3^H] PK11195 binding in the spinal cord seems to occur in microglial cells. TSPO is strongly expressed in activated microglia (Benavides et al., 1983; Moynagh et al., 1991; Itzhak et al., 1993; Park et al., 1996; Karchewski et al., 2004; Hernstadt et al., 2009; Varga et al., 2009). Microglial TSPO requires a particular attention due the key role of this cell type in the initiation and maintenance of chronic pain states (Tsuda et al., 2005). The recruitment of TSPO signaling is also of interest while dealing with pain models associated with lesions during the process of recovery. For example, regulation of the expression of TSPO and of their endogenous ligands have been well studied during rat sciatic nerve degeneration and regeneration (Rupprecht et al., 2010). After nerve freezing lesion or chronic denervation, a clear over-expression of TSPO and octadecaneuropeptide (ODN) is observed in Schwann cells and macrophages, suggesting their crucial role during regenerative processes (Lacor et al., 1996). Axonal injury-dependent induction of TSPO has been also observed in small-diameter adult rat primary sensory neurons (Karchewski et al., 2004).

## Endogenous control of allopregnanolone synthesis and analgesic function

Many endogenous ligands of TSPO have been identified and are described in detail in a recent review (Rupprecht et al., 2010). If their binding characteristics and neurosteroidogenic activities are well understood, very little is known regarding their role in brain functions and pathologies (Rupprecht et al., 2010). Cholesterol and porphyrins are important endogenous ligands of TSPO and display nanomolar to micromolar affinities, respectively. Endozepines, discovered at the end of the 80 s, are peptidergic ligands capable of displacing the binding of classical benzodiazepines at their GABA_A_R binding sites (Costa and Guidotti, 1991). The endozepine diazepam-binding inhibitor (DBI) and its metabolites were later shown to stimulate neurosteroidogenesis after binding to TSPO (Papadopoulos et al., 1991; Do-Rego et al., 1998). Few reports are available suggesting their implication in pain. An antinociceptive effect of DBI on thermal and mechanical thresholds has been reported after intrathecal or i.c.v. infusion of DBI (Wang et al., 2002). In line, i.c.v. injection of a bovine endozepine potentiated morphine analgesia in mice (Chen et al., 1991). In apparent contradiction, the DBI-derived ODN was shown to increase aggressive interactions in mice and, surprisingly, to reduce defeat-induced analgesia (Kavaliers and Hirst, 1986). In summary, these rare reports suggest a role for endogenous TSPO ligands in the control of nociception. The real expression levels of endogenous TSPO ligands, their cellular localization and changes in pain pathologies are, however, still largely unknown. This is in sharp contrast with TSPO expression which has been found to be increased in several neuropathologies including in pain models.

Beside TSPO ligands, we showed that oxytocin, a neuropeptide released by hypothalamic neurons projecting onto spinal nociceptive neurons, is exerting its long-lasting analgesic effect by stimulating the production of AP (Juif et al., 2013). Apart from its fast antinociceptive action in the spinal cord which increases GABAergic inhibitory transmission and reduces neuronal excitability (Breton et al., 2008, 2009), the tonic activation of spinal oxytocin receptors in inflammatory pain states also stimulates AP synthesis via ERK (for extracellular signal-regulated kinase) signaling pathways, resulting in the potentiation of GABAergic inhibition and limitation of pain symptoms (Juif et al., 2013). It is not excluded at this stage that other descending inhibitory controls may also exert their antinociceptive action by modulating neurosteroid synthesis, but this remains to be demonstrated. Another example is related to substance P secretion by nociceptive primary afferents which may inhibit the production of AP through its NK1 receptor, as previously demonstrated (Patte-Mensah et al., 2005). Taken into account this result, we may speculate the diffuse AP-based analgesic system to be under the control of a balance of stimulating and inhibiting neuropeptides in the spinal cord. This hypothesis requires to be tested in order to identify the most promising peptides for future therapeutic interventions.

## Conclusive remarks

In conclusion, there are now several convergent experimental evidences demonstrating that the local production of AP-like steroids in the nociceptive system constitutes a diffuse analgesic system. The development of numerous TSPO ligands capable of stimulating this system is on the way, but little is known regarding other possible physiological stimulators. In line with this idea, neuropeptides could play a key role.

## Conflict of interest statement

Pierrick Poisbeau is currently collaborating with Biocodex laboratories to decipher etifoxine action.
